# Managing and treating intraocular retinoblastoma

**Published:** 2018-06-03

**Authors:** Ashwin Reddy, Mukesh Jain, Vikas Khetan

**Affiliations:** 1 Lead Clinician for Ophthalmology and Retinoblastoma Services: Royal London Hospital, London, UK.; 2 Research-cum-Clinical Vitreo-Retinal fellow: Sankara Nethralaya, Chennai, India.; 3 Senior Consultant: Sankara Nethralaya, Chennai, India.

**In order to improve the survival rates of children with retinoblastoma, a collaborative and multidisciplinary approach is essential, as is a listening ear for parents who may struggle with the difficult decisions facing them**

**Figure:**
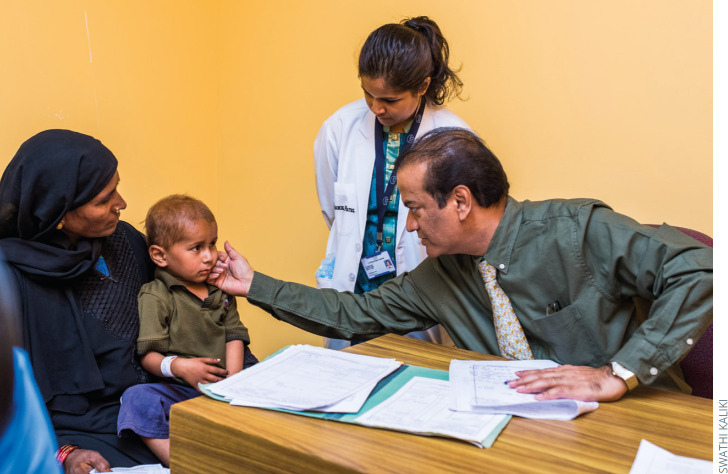
It is important to consider the whole child, not just the eyes. INDIA

Enucleation remains the mainstay of treatment for advanced intraocular retinoblastoma. This is particularly important in unilateral retinoblastoma in groups C, D and E (Table 1) in low-resource countries, where follow-up may be poor. Acrylic implants and shells/prostheses may cost less than US $10 each and, if available, will ensure that the social and emotional welfare of the child and parents are catered for.

## The importance of listening to parents

Doctors who deal with retinoblastoma and enucleation regularly can forget how terrifying it all is for the newly diagnosed family. Taking time to truly listen to parents and address their concerns are an essential part of counselling about treatment and rehabilitation options. Surgery can save a life, but so too can a quiet, listening presence and responsive, empathetic guidance that meets the family in their place of pain and helps them to find a way out of it. The parents of a child whose life has been saved by unilateral enucleation may also be helpful in counselling other families who may think about refusing enucleation due to fear about the outcome.

*With thanks to Abby White of World Eye Cancer Hope for her contribution on counselling parents. **www.wechope.org***

**Figure:**
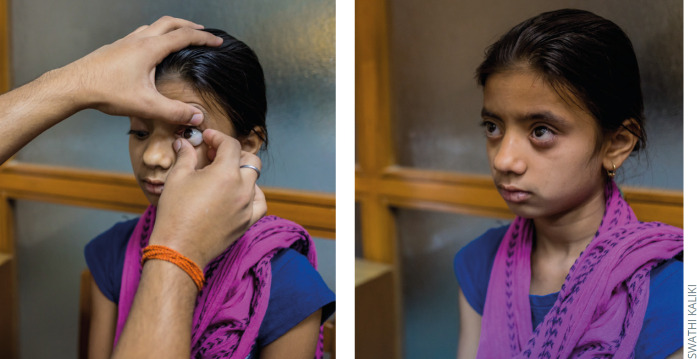
Good prostheses ensure that the social and emotional welfare of the child and the parents are catered for.

## Multidisciplinary approach

In order to reach - or maintain - high survival rates of over 95%,^1^ it is essential that there is a collaborative multidisciplinary approach with a trained pathologist and a paediatric oncologist. Otherwise, high-risk features such as massive choroidal invasion and/or retrolaminar optic nerve invasion will not be detected and the child could still die from metastases (secondary spread of the tumour), despite enucleation.

## Systemic chemotherapy

This may be given systemically as first line treatment for children with intraocular retinoblastoma in groups B, C and D (Tables 1 and 2), particularly those affected in both eyes. Regular follow-up is *essential.* If systemic chemotherapy is given, with additional focal therapy (see below), enucleation can be avoided in over 95% of eyes with groups B or C retinoblastoma.

## Focal therapy

Focal therapy includes transpupillary thermotherapy (TTT), laser photocoagulation, cryotherapy, and plaque radiotherapy. Of these, laser and cryotherapy are likely to be available in low-income countries. All of these can be used either alone (in infants with retinoblastoma identified as being in Group A and possibly Group B), or after initial systemic chemotherapy in Groups B, C & D retinoblastomas. Focal therapy works best for small tumours (less than 5 mm), or recurrences with no associated vitreous and/or subretinal seeds.

### Laser photocoagulation

Laser treatment is likely to be more readily available than TTT as the laser used for diabetic retinopathy in adults can be modified and used as an indirect laser for treating retinoblastoma. Treatment involves application of argon 532/810 mm laser (above 65 °C) either directly on the tumour, or in a ring-like fashion around it to coagulate the feeding blood vessels, leading to ischaemic tumour damage. In the next 2–3 sessions, the tumour is repeatedly covered with laser burns. Complications include vitreous seeding, vascular occlusions, pre-retinal fibrosis and associated retinal traction and vitreous haemorrhage.

### Cryotherapy: Anterior/peripheral small tumours

This involves the application of sub-freezing temperatures (down to −90 °C) directly to the tumour mass, resulting in damage to the vascular endothelium with secondary thrombosis and infarction of the tumour. Tumours are typically treated by triple freezethaw technique through the conjunctiva in two sessions with a 3-weekly interval (Figure 1). Complications include lid oedema, conjunctival chemosis (swelling), serous retinal detachment, vitreous condensation (which can result in vitreous haemorrhage) and tractional retinal detachment.

### Plaque therapy: Larger tumours or areas of relapse

Radioactive plaque brachytherapy can be used for tumours with basal diameter and height less than 16 mm and 8 mm respectively. Iodine (I^125^) and Ruthenium (Ru^106^) radio-isotopes are most commonly used. Gold plaques carrying radioactive seeds are sutured to the base of the tumour, to provide 40 Gray (Gy) to the tumour apex over a period of 2–4 days, and the plaque is then removed. Major complications include cataract, radiation retinopathy or papillopathy, optic neuropathy, scleral necrosis and keratopathy.

**Table 1:** Treatment of intraocular retinoblastoma

International Intraocular Retinoblastoma Classification (IIRC)		
	Definition	Treatment
**A**	Small tumours <3 mm outside macula	Focal treatment. If no focal treatment available either: Send to a site with focal treatment or if no focal treatment is available in country please seek expert guidance
**B**	Bigger tumours >3 mm or Tumours in the macula or Tumours with sub-retinal fluid	Focal treatment with or without (+/−) systemic chemotherapy up to 6 cycles
**C**	Localised (within 3 mm from the tumour) vitreous or sub-retinal seeds	**Unilateral:** Enucleate **Bilateral:** Attempt ‘Second Eye’ Salvage: Systemic chemotherapy 6 cycles +/− focal treatment
**D**	Diffuse (> 3 mm away from the tumour) vitreous or sub-retinal seeds	**Unilateral:** Enucleate **Bilateral:** Attempt ‘Second Eye’ Salvage: Systemic chemotherapy +/− focal treatment. IF EYE SALVAGE FAILS: enucleation.
	**If Enucleated** look for: **High Risk Histopathological Features:** Retrolaminar optic nerve involvement Choroidal Invasion >3 mm	**Post Enucleation:** If low risk histopathological features present - no further treatment If high risk histopathological features present: 6 cycles of chemotherapy
**E**	Any of the following: • tumour touching the lens • neovascular glaucoma • tumour in the anterior chamber • opaque media due to vitreous haemorrhage • aseptic orbital cellulitis • phthisis bulbi.	Enucleation. If low risk - no further treatment. If high risk histopathological features present (as for Group D): 6 cycles of chemotherapy

**Table 2:** Standard dose systemic chemotherapy given every 3 weeks for Intraocular Rb

Drug	Dose: Mg/m^2^	Rate of infusion	Diluent
Vincristine	1.5mg/m^2^ body surface area (BSA D1) (*to a max. of 2mg/dose*)	Slowly over 10 minutes	Not less than 10 mls of 0.9% NaCL **Note: Risk of extravasation**
Etoposide	300 mg/m^2^ BSA D1	4-hour infusion	0.4mg/ml in 0.9% NaCI **Note: Rapid infusion will lead to hypotensive crisis**
Carboplatin	600 mg/m^2^ BSA D1	1-hour infusion	0.5mg/ml in D5% or DNS
**Requirements before each cycle**			
ANC > 1; Platelets > 100; Check that Hb, Renal profile, LFT's and Magnesium levels are adequate			
**Age**	**Dose modifications**		
< 6 mth	Give 50% of the dose for each drug		
6–12 mths	Give 75% of the dose for each drug		
12+ mths	No modification		

**Figure 1a:**
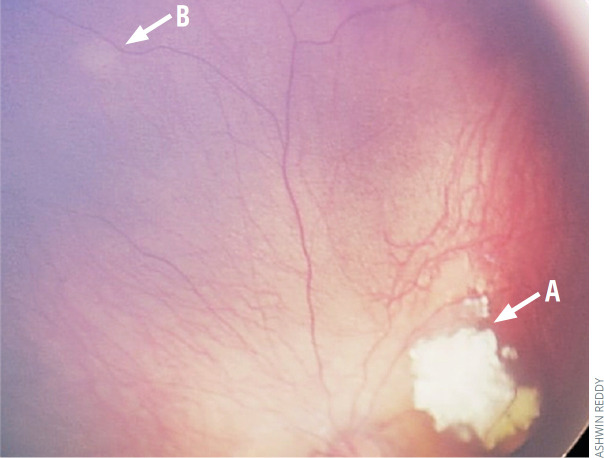
A child with a Group D retinoblastoma in one eye has been treated with systemic chemotherapy and the main tumour has calcified (A). However, a new tumour (B) has formed peripherally/anteriorly which was detected at a regular follow-up visit. Regular follow-up is essential as new tumours can be difficult to detect in the early stages.

**Figure 1b:**
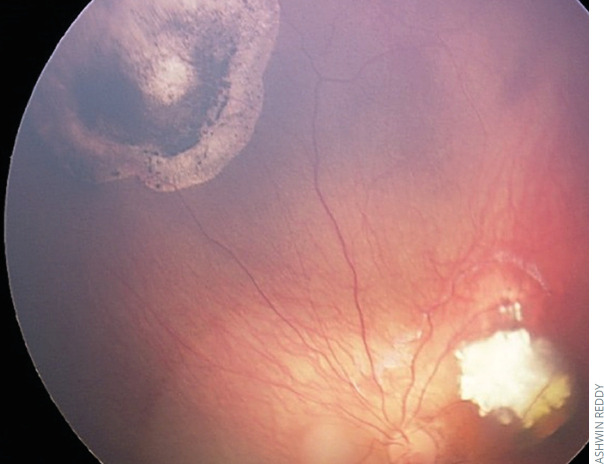
The new peripheral tumour has been treated with two sessions of triple freeze-thaw cryotherapy, spaced 3 weeks apart, resulting in a flat scar. If the tumour had not been detected during a regular follow-up visit, the child would have returned with an untreatable tumour in the eye which would have required enucleation and the child would have been at risk of metastasis and death.

## External beam radiation therapy

External beam radiation therapy (EBRT) for the treatment of retinoblastoma has decreased drastically due to the increased risk of second non-ocular malignancies, particularly in infants under the age of 12 months who have germline retinoblastoma. However, it does have an important role in children with extraocular retinoblastoma in the orbit (see page 19).

## Intravitreal chemotherapy

The vitreous contains no blood vessels, therefore drug concentration from systemic chemotherapy is less effective. Melphalan and topotecan (either singly or in combination) are the chemotherapeutic agents that have been given by direct intravitreal injection.^2^

Intravitreal injection should not be given:

- if there is tumour at the site of the injection
- if tumour extends to the ciliary body
- if there is a bullous retinal detachment or vitreous haemorrhage which obscures the view of the vitreous and retina.

An ultrasound biomicroscope (UBM) or direct vision may be used before treatment to assess the injection site for tumour.

A small diameter needle must be used (30G or smaller). The site of injection should be 3 mm from the limbus into the pars plana. We suggest using a triple freeze-thaw cryotherapy at the injection site as the needle is withdrawn. The eyeball is then gently jiggled with forceps to distribute the drug evenly throughout the vitreous.

The recommended dose of intravitreal melphalan is 20–30 microgrammes for a maximum of 6 injections over 2–3 months, depending on the distribution and extent of vitreous seeds and response to prior injection. Eye salvage rates have significantly improved as a result of this treatment.

## Intra-ophthalmic artery chemotherapy

Although not widely available in low-resource countries, direct treatment of the eye via intra-ophthalmic artery chemotherapy has overtaken the use of EBRT for retinoblastoma once systemic chemotherapy and focal therapies have been exhausted. This treatment should be used with caution as first-line treatment for unilateral advanced retinoblastoma (Groups D and E) as they may metastasise. This is particularly relevant if follow-up is poor.

**Figure 2:**
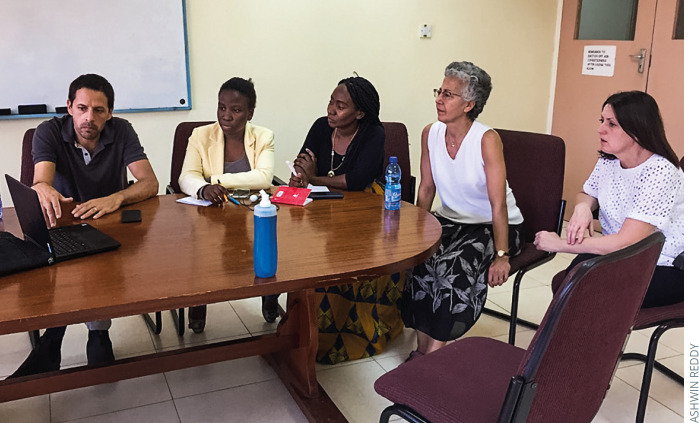
A multidisciplinary team discussion in Malawi
